# A Treatise on the Irregularity of the Teeth and Their Correction

**Published:** 1891-01

**Authors:** 


### Bibliographical.

A TREATISE ON THE IRREGULARITY OF THE TEETH AND THEIR CORRECTION, including, with the author's practice, other methods. By John Nutting Farrar, M. D., D. D. S., volume 1st, New York City, 1888. The first volume of this extensive work of Dr. Farrar has made its appearance, and has fully and completely met the anticipations of all those who were cognizant of its preparation. There is no other work on the subject that in any manner equals or excels this great treatise. The author in his preface remarks: "I have aimed to make my views especially clear, not only to correct current erroneous impressions, but also to place the results of my experience before the profession to whose welfare and progress I have striven to contribute during the best years of my life.

"When I began to practice, principles, methods, and even suggestions in regard to regulating teeth were meagre. The apparatus in use was crude, clumsy, uncleanly, and painful. Invention, to me, has always been a diversion, not a labor, therefore I found enjoyment in this occupation; and when I succeeded in making, among various instruments, devices that seemed to be an improvement on the established forms of regulating apparatus, I thought it no more than right to describe them for the benefit of others." In the first chapter the author also remarks: "The importance of the observance of the physiological law which governs tissues during a movement of the teeth (by means of art), the object being to prevent pain, to insure this result the pressure by which the movement is to be effected should be under the control of the patient, a requirement which implies the use of instruments capable of being operated and adjusted at will."

For convenience, the work is divided into three volumes (the two additional volumes soon to follow the first,) and, not including those of the third volume, will be illustrated with nearly 2,000 engravings.

The first volume relates to the history and etiology of Irregularity: The Basal Principles of Regulation; Nomenclature;

Principle of Construction of Apparatus; Retaining Devices; Laboratory Rules for Manufacturing Devices; Application of Force; Eruption; Antagonism; Interdental Spaces; Correction of Irregularities by Grinding, and by Extraction.

The second volume contains the Classification of Irregularities, and the Various Methods of Treatment for Correction; Straightening Teeth to Line; Turning and Elevating Teeth; Widening and Enlarging the Dental Arch; Correction of Protruding Teeth; Miscellaneous Suggestions, Practical and Theoretical; and lastly, Æsthetics of Dentistry. . . . The third volume is wholly pictorial, being an object index of all mechanisms described in the other volumes.

The third volume refers to "History", and quotes the labors of Dr. Van Marter, of Rome, on the work of the Etruscans, a race which preceded the Romans; also extracts from Dr. H. Eames' "Russian Antiquities," with illustrations and descriptions of instruments from Pompeii, made during a visit to the Museum at Naples. It also contains the "History of the Use of the Screw" in regulating teeth.

The third part of this volume refers to many interesting subjects, among others, The Influences of Evolution upon the Jaws; Premature Extraction of Deciduous Teeth; Power of Heredity· Influences of Insanity and Idiocy upon the Teeth.

"Upon the Question of Hetezogeneousness in the Mixture of Races," the author concludes that "there is an underlying power, a law that tends to evolve order out of confusion, not only in mental but also in bodily forms. If study of this law should find a place in the science of dentistry, it would contribute to an education that would lead to broader views." In this third part the "Philosophy of the Author's System," is given in a manner that reveals the basis of the work. 1. That in regulating teeth the force should not exceed the bounds of physiological functions. 2. That the plan of moving teeth by elastic materials, though practical, if kept under perfect control, is so difficult to manage that it often leads to pain and inflammation, and is sometimes dangerous to the future usefulness of the teeth, while a properly-constructed apparatus, intelligently operated by means of screws, insures beneficial results without pain or nervous exhaustion. 3. That if teeth are moved by absorption through the alveolar process about 1-240 of an inch every morning, and the same distance in the evening, no pain or nervous exhaustion follows. 4. That while these tissues will allow an advance of a single tooth at this rate (1-240 of an inch)twice in twenty-four hours without exceeding physiological functions, if a much greater pressure be made the changes will become pathological.

All of which renders this great work one of the most valuable additions of the present age to dental literature.

